# Unreliable numbers: error and harm induced by bad design can be reduced by better design

**DOI:** 10.1098/rsif.2015.0685

**Published:** 2015-09-06

**Authors:** Harold Thimbleby, Patrick Oladimeji, Paul Cairns

**Affiliations:** 1 College of Science, Swansea University, Swansea SA2 8PP, UK; 2 Department of Computer Science, University of York, York YO10 5DD, UK

**Keywords:** number entry, human error, dependable systems, evaluating user interfaces

## Abstract

Number entry is a ubiquitous activity and is often performed in safety- and mission-critical procedures, such as healthcare, science, finance, aviation and in many other areas. We show that Monte Carlo methods can quickly and easily compare the reliability of different number entry systems. A surprising finding is that many common, widely used systems are defective, and induce unnecessary human error. We show that Monte Carlo methods enable designers to explore the implications of normal and unexpected operator behaviour, and to design systems to be more resilient to use error. We demonstrate novel designs with improved resilience, implying that the common problems identified and the errors they induce are avoidable.

Science is a way of trying not to fool yourself. The first principle is that you must not fool yourself, and you are the easiest person to fool.

—Richard P. Feynman [1, ch. 4]

## 1. Introduction

Number entry is often performed as a ‘simple’ subtask within a bigger task. For instance, using a calculator typically requires entering a series of numbers and operators. Unnoticed errors while entering the numbers would result in an error in the calculation. To the user who needs to use a calculator and therefore has no precise expectation of the result, this error is likely to go undetected and escalate higher up into the user's workflow or subsequent tasks.

As users of interactive systems, we have little idea how much our unnoticed errors introduce inaccuracy or other problems. Our laboratory work [2] suggests about 3.5% of numbers we enter (on conventional numeric keyboards) are wrong and *we do not notice that they are wrong*. Consequently, designing interactive systems to reduce the rate of unnoticed use errors is a worthwhile goal. Unfortunately, the same human error problems—errors happen and remain uncorrected because we are largely unaware of them—beset designers and manufacturers too: they do not know some designs are defective and cause problems for users. Finally, purchasers are unable to compare and choose more dependable or safer equipment when it is available.

When we enter numbers into a system or piece of equipment, some numbers will be wrong because we make typing slips or other errors. Numbers will remain wrong if we do not notice they were wrong. We may use various techniques, such as entering lists of numbers twice (e.g. checking totals are the same) or entering checksums to help detect possible errors.

If we notice errors as we type in numbers, we typically use strategies like pressing 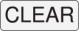 or 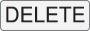 keys to help to correct the errors.

Unfortunately, as this paper shows, common defects in system design can leave corrected numbers *still* wrong. Additional unnoticed errors can occur during the error correction process. If we do not notice the ‘corrected’ numbers are still wrong (perhaps wrong in different ways), then the numbers will remain wrong even though we think they are correct because we corrected them. To our knowledge, this paper is the first to report and analyse this issue.

The problems we address in this paper can be found widespread in everyday products that have been manufactured and used for years. Awareness of these potentially critical problems is evidently very low. In this paper, we show how to address the problems and how to evaluate their impact. Further, we show that the problems are avoidable, by better production processes and by more careful purchasing of better products.

We are worried about the scale of preventable errors induced by poor system design, and by the possibility that users and operators are being blamed for errors that are not of their making. The problems are particularly worrying in areas such as healthcare, where incorrect numbers may lead, for instance, to incorrect drug doses and patient harm. In other areas, such as economics, finance and science, unnoticed incorrect numbers may remain unnoticed and affect policy or mislead further work.

Because the scale of this avoidable problem is so surprising, this paper includes a review of the background on human error and the nature of number entry. Section 2 of this paper explores the cultural context that has allowed poor design—the absence of applied science—to become so common. Section 3 then presents our methodology, and finally §4 provides discussion and conclusions drawing on the results of our investigations.

### 1.1. Our previous work

The present paper develops our work reported in previous papers.

Most recently, in [3], we surveyed numeric user interfaces and showed that many are poorly designed and implemented. We showed how to formalize interaction using Hoare Triples, an approach that allows rigorous reasoning about design correctness, with all the usual benefits of formal methods but applied to user interface design. We have shown that formal methods can detect design errors [4]. However, formal methods do not in themselves help make value judgements about which designs are better—they help developers to more reliably implement whatever they wish to implement. Therefore, in this paper, we show how to measure and quantify design issues, using Monte Carlo methods. We will present results from measuring the performance of several designs.

In [5], we showed that simulating a user by a stochastic process can estimate the safety of numeric user interfaces, specifically by counting ‘out by 10’ numeric errors. We provided evidence to substantiate our claim that failings in user interfaces are ‘ubiquitous’. We showed that modifying user interfaces to conform with well-known standards would make them safer.

In much earlier work [6], we showed how a Markov process can be used to evaluate the quality of user interfaces. This approach (which we did not then apply to numeric user interfaces) has the advantage that it avoids many assumptions about usability—the Markov process ‘knows nothing’ about design assumptions, and thus the technique is very powerful in identifying potential design issues that may have been overlooked. Markov models are technically hard to use, so in [7] we showed how Monte Carlo methods can perform comparable analyses. (Using Markov models requires more mathematical skill; using Monte Carlo methods is much simpler but requires more computer time.)

### 1.2. A new approach

We propose a Monte Carlo approach to help designers avoid user interface design problems in the first place, as well as to help users (e.g. during procurement) choose better designs.

Because the approach uses Monte Carlo methods (which we describe in more detail below), it can be applied to final implementations, and therefore can help detect implementation bugs after systems have been completed: it is not just a formal technique that is used in requirements or specification. In particular, it can help find design defects that were not anticipated during specification and which otherwise might therefore remain in a system as ‘unknown unknowns’. Monte Carlo methods are easy to understand and use, and have none of the daunting problems of conventional formal methods, which can create other sources of design problems.

In areas like hospital procurement, when critical systems may be procured for widespread use, basic Monte Carlo testing could provide large improvements at the organizational scale. More broadly, by developing a clear way to measure trade-offs this paper raises awareness of these ubiquitous design problems. We also show how they are preventable.

## 2. The cultural context

### 2.1. Human error

Errors are ubiquitous. Accidents happen because we do not notice errors soon enough to manage or mitigate them—errors are frequently noticed only in hindsight, often after an inquiry into an accident. If an error can be noticed and repaired fast enough, it need not lead to harm, except as might be occasioned by any delay in its repair. Unnoticed errors, then, lead to inaccuracy, and sometimes to adverse or harmful consequences. In general, errors themselves are not the problem, but the unwanted consequences of unrepaired or unsuccessfully repaired errors are.

In many contexts, systematic learning is instigated after noticed harm, for instance by performing an after-action review or root cause analysis to explore the factors leading to the harm. The systematic exploration of causes has to stop somewhere, typically stopping at a human operator (user, practitioner, scientist, pilot, etc.), concluding that ‘human error’ is the root cause [8]. System defects further encourage blaming the operator as the logs or records may misrepresent the operator's actions: if the design mismanages an error repair, the mismanagement is recorded as if it is what the operator actually instructed the system to do.

Finding out what went wrong can fuel a spiral of delay, litigation, secrecy and denial. It is more productive to think about how to help ensure things go right more often in the future [8]. To do so requires a different perspective: how to change the system, and how to know whether and to what extent proposed changes affect safety—fuelling a positive spiral of action, innovation, disclosure and evidence-based improvement [9].

In science more generally there is low awareness of routine error and its consequences, with more emphasis on fraud and incompetence. *Nature'*s editorial comment [10] that ‘underlying these issues, often, is sloppiness, whether in the handling of data, in their analysis, or in the inadequate keeping of laboratory notes. As a result, the conclusions of such papers can seem misleadingly robust’. To this list, the present paper adds misleading sloppiness in the design of the equipment or systems the authors of these papers are relying on to do their research.

In most systems, there are interrelated agents who manage or are affected by error (table 1). Although these roles do not always divide neatly into different individuals (for example, somebody may be injured by a system they designed for their own use), there is a crucial difference between *operator* and *designer*.

**Table 1.** Terminology used in this paper. The table makes clear that the designer has responsibility both at the blunt end and at the sharp end. (In a sense, the regulators, procurers and managers are all designers, since they specify or choose from a set of designs, which itself is a design activity.)

blunt end 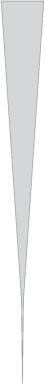 sharp end	regulator	the organization that specifies high-level design rules and procedures (such as ISO 9241, ISO 19471, etc.)
	designer	the person or persons who design, create or program the system. Designers are typically remote, as in manufacturers or their sub-contractors. In this paper, we are particularly concerned with designers of interactive systems
	system	the environment in which the operator works. The system includes the devices as well as the standard operating procedures, training and other people. (This paper is particularly concerned with the human interface of automated parts of the system.)
	procurer	people who choose designed (manufactured, programmed) products and assemble them into local systems
	manager or supervisor	people who are responsible for and devise rules within which operators work. Managers typically set requirements for designers
	team	in resilient organizations [11], the operator is seen as working within an effective team; other people help the operator avoid, monitor and mitigate error
	operator	the person ‘at the sharp end’ who is normally (but not always appropriately) considered responsible for outcomes
	device	the part of the system that physically causes the incident; for example, the operator may have pressed a button on the device, but the device actually caused the harm
	victim	the person or persons immediately suffering from the consequences of unmanaged or inadequately managed error
	second victim	operators or others who suffer indirectly, for instance from depression or inappropriate line management response [12]

Operators work under pressure to manage concurrent, real-time task demands, and they are typically unable to walk away from their tasks to ‘time out’ and reflect. They work under an unavoidable efficiency–thoroughness trade-off (ETTO) [13]: the more they accommodate to the demands of the tasks, the less they can be thorough anticipating, detecting or managing error. On the contrary, designers can and should be thorough designing systems that are resilient to error—*their* tasks are not constrained by real-time or other situational issues (except for arbitrary marketing or manufacturing deadlines, that arguably should not trump design quality considerations). For example, the operator of an infusion pump might be an anaesthetist with a patient dying right in front of them if they do nothing; whereas the infusion pump manufacturer had years to refine the design of the pump the anaesthetist is now operating. Designers should therefore tilt the ETTO principle in favour of thoroughness for the benefit of operators. Unfortunately, like operator errors, design errors occur because designers do not notice them.

Designers fail to notice errors for largely the same reasons as operators do, namely loss of ‘situational awareness’ [14]: design is hard enough already without having to worry about unlikely operator error. Design errors remain as ‘latent conditions’ [15] that may induce operator error, fail to warn operators of error, or exacerbate operator attempts to recover from error. Although formal methods are increasingly used to improve the reliability of programs, it is only very rarely applied to the user interface. The user interface ‘just provides numbers’ and the program handling those numbers may be correct, but the user interface has not been formalized [3]. Designers need new methods to identify design errors and to evaluate their impact—and to help design more reliable systems.

### 2.2. Motivating problems

The introduction provides context for our research. We are particularly motivated by five observations together painting a tragic picture:

—Systems in widespread use have subtle design defects [5,7,16–19]. We give concrete examples throughout this paper.
—90% of medical devices are released onto market without testing [20]. Software-related recalls of medical devices are increasing [21].
—Preventable death in US hospitals is estimated to be approximately 440 000 per year [22]—scaled by UK : US population, that is some 87 000 preventable hospital deaths in the UK per year. Severe harm is estimated at 10–20 times higher. Unfortunately, we do not know what proportion is design-related, though user programming errors involving tasks such as entering and modifying drug dose parameters in a *single* hospital infusion pump model were estimated to contribute to 65–667 US deaths per year [23].
—When patient harm occurs, the professionals involved are also harmed [12], more so if attribution of blame is unjustified. This occurs as investigators are largely unaware whether (and, if so, how much) error is induced by poor design of devices.
—There is very little applicable science in the area. There needs to be an effective way to start to measure and scope the problem, in particular to help drive informed improvement.

It might seem that our emphasis on medical user interfaces makes this paper more specialized than it is. On the contrary, the user interface defects reviewed here occur in every type of user interface, but especially for medical systems one might have expected greater care to be exercised in their design and requirements, since the consequences of failing to do so directly costs lives. There is no evidence that medical systems are designed any better; indeed the routine confidentiality surrounding medical system design ensures that rigorous evaluation (whether needed for research or for informed device procurement) and public discussion on quality are much harder than they need be. The confidentiality plus the variation in design across brands tends to lock operators into using, or wanting to use, specific types or makes of device: different, possibly even safer, user interfaces will feel more awkward in hard-to-quantify ways.

For all these reasons, we need to help designers and developers avoid or reduce the problem and its impact, help procurers choose between designs in an informed way, and help operators adopt strategies to reduce errors on the systems they have to use—and help them identify, articulate problems, complain and resist having to use defective systems. We have to help investigators and reporters understand the central role of poor design in causing incidents: does such ignorance warrant a newspaper headline calling a nurse ‘blundering’ [18,24]?

### 2.3. Repairing error, and problems of defective design

Skilled typing (how most computers systems are used) involves two nested mental processes, an ‘outer’ one involved with the intention to type and an ‘inner’ one involved with the lower level actions to physically type [25]. The lower level process can detect errors and repair them by, for instance, pressing a delete key. Repair can be achieved by skilled typists without conscious awareness at the higher level. Incorrect implementation of the delete key is therefore unlikely to be noticed, which in turn may lead to further errors.

Delete keys for repairing errors are widespread. On many devices (typically mobile devices, but also simulations of devices on PCs, such as ‘desktop’ calculator applications), neither the decimal point nor the delete key work correctly in a way that can be reliably learned by the lower level repair processes. On many devices, additional decimal points are ignored, so deleting a second decimal point misleadingly deletes all decimal points. On some devices, the delete key ignores decimal points altogether and only deletes digits, so 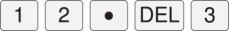 (which the operator might think would be corrected to 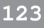) becomes treated as 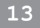.

Repeatedly pressing or how long a key is held down may change its behaviour (e.g. pressing 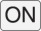 twice or holding it for several seconds switches some devices off): on such systems exact key press timings need to be recorded. Correctly logging user interaction is particularly important on user interfaces with touch screen technology where user input might be through gestures, or multiple contacts on the screen. On many systems, then, logs purporting to record operator actions are misleading, making it impossible to distinguish between operator errors and repaired errors the system defectively corrects.

Some number entry design problems of the sort we are concerned with are illustrated by the widely available Apple iPhone calculator (checked on iOS versions 7.1.2 through 8.4, 2015) as follows:

—keying 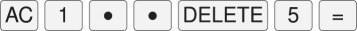 gives 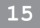, 10 times higher than intended;^1^
—keying 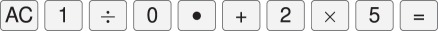 gives 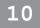, when it should be reported as an error the calculator detects (see table 2 for step-by-step details);
—keying 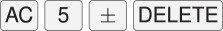 gives 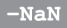, a nonsense result (NaN means ‘not a number’ and is the consequence of an internal design error that should not have become visible to the user [26]); and
—if the user has already entered part of a number, say, 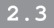 pressing 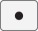 will keyclick normally yet do nothing.

**Table 2.** Detecting error on the Apple iPhone calculator. We illustrate the problem with division by zero in the example where the operator intends to calculate 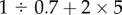 but omits the 7 in error. Division by zero is detected, and 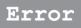 is displayed, but the operator continues, and finally reaches a display that appears to show that 10 is the correct answer to the calculation (the correct answer is 11.4285714 to the precision of the iPhone). A more dependable calculator would display 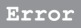 *continuously* until 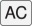 is pressed or the operator otherwise indicates they have recognized the error.

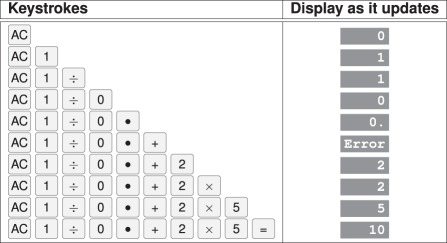

Such design defects are surprising, as Apple is widely recognized as the leading manufacturer of high-quality, easy-to-use products. Calculators are not complex, and in principle they can be rigorously engineered to be reliable.

The iPhone number entry shows at most one decimal point, which is unlike most calculators which always show exactly one decimal point. On these calculators, pressing 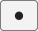 never has any visual effect, even though many provide keyclick feedback which normally implies the key did something.

Further number entry design errors in the iPhone and other manufacturers’ similar products have been noted elsewhere [16,18,27,28].

### 2.4. Why do problems persist?

This paper exhibits a wide range of basic defects with the design of number entry user interfaces, yet these are mature user interfaces that have been deployed very widely and from respected manufacturers.

Neither manufacturers nor operators are noticing these basic problems nor trying to fix them, even for when systems are used, as calculators routinely are, in safety- and mission-critical applications. If nothing else, it is evident that dependability (safety) and ease of use are different things, and when aiming for dependability, ease of use is deceptive—if something looks and feels nice, it may not help the operator be safe and effective

The question is begged, why do the problems persist?

A range of possible answers is presented in appendix A. The answers show how low awareness leads to persistent low awareness and then to inaction. Even with best practice using formal methods, it is not possible to formalize design principles of which one is unaware.

Our previous work [29] studied a deeper problem: not only are the user interfaces for number entry defective, but the programming languages that implement them are defective too: many of the issues we discuss in this paper apply not just to interactive user interfaces but to numbers *in* programs. Even motivated programmers may have a huge job ahead of them if they wish to implement dependable user interfaces.

## 3. Towards solutions

To start to address the problems raised above in §2, we propose a simple, rigorous process to reveal and quantify important variation in design—variation that usually goes unnoticed, with the result that poor design choices are often made. The approach introduced in this paper of quantifying aspects of user interface quality (here, applied to numeric user interfaces) will help break some of the deadlocks to progress.

Put briefly, human error occurs because we are unaware of facts that if they had been properly considered would have changed what we did. Unfortunately, the nature of human cognition ensures it is not possible to arbitrarily increase awareness—to perform a task requires concentration, which leads to loss of ‘situational awareness’ and inevitably there is a trade-off between performing a task well and being aware of the wider environment [13]. While we might like to just increase awareness, in practice it is not so straightforward.

Instead, we prefer to think of error being dependent on ‘vulnerability’. If we imagined awareness and vulnerability as simple probabilities, then
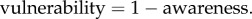


However, the differences are more profound: focusing on awareness, the word itself seems like it is the user's or operator's own problem to be more aware; while focusing on vulnerability, it is more clearly the system's responsibility to create a less vulnerable environment. This in turn implies the designer should be more aware—developing systems that help reduce and manage vulnerability.

### 3.1. Safety metrics

We define **vulnerability** *v* as the conditional probability an operator does not attempt to repair a keying error,
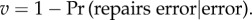
Here ‘repairs error’ means the operator attempts to repair the error in any normal way; hence harm occurring when *v* = 0, when the operator always repairs errors, is caused by design defects—repairing an error correctly may fail on some devices. Monte Carlo experiments make it easy to simulate human behaviour with any *v* and with any distribution of error probability.

We define **risk** *r* conventionally as the expectation of harm. Various metrics can be used depending on the task: counting ‘out by *f*’ errors for quantities that have to be within a tolerance factor *f* but do not need to be exact; counting over-doses but ignoring under-doses; or measuring the expectation of the ‘out by’ ratio. A simple metric is clearest for this paper: we take harm to be 1 if the intended number and the entered number are different, 0 if they are the same. This is a proxy for harm for tasks like entering passwords, credit card IDs, patient IDs, all of which have to be exact or will fail.

As vulnerability increases, for any reason, we would expect risk to increase (other things being equal). We therefore introduce **risk ratio**, the ratio of risk to vulnerability, *r*/*v*.

Ideally, risk ratio should be as low as possible. Figure 2 vividly illustrates how risk ratio highlights two common but poorly performing designs, contrasting them with more dependable alternatives.

As operators or training and procedures reduce or attempt to reduce vulnerability it is important that risk ratio also decreases (and certainly does not increase)—otherwise the improvements will be counter-productive, made so by defective design.

It is possible to further refine these concepts, but this is unnecessary for our purposes. Indeed, we suggest that having more complex definitions of vulnerability or risk would tend to obscure some of the issues that remain obvious with simple definitions.

### 3.2. Monte Carlo methods for numeric input

Performing experiments with human operators that last long enough to encounter enough unnoticed errors to establish whether purported design failings are statistically significant is very time-consuming to undertake, and is certainly excessively time-consuming to perform repeatedly as a design is iteratively improved.

Instead, in this paper, we run Monte Carlo experiments on user interfaces. The Monte Carlo experiments simulate human typing, involving both error and error repair.

Probabilistic methods have previously been used to find input that crashes programs [30], but, apart from our own work [5,31], building on methods to assess usability [6,7,19], they have not been used to assess safety or accuracy. The present paper is the first to consider operator error correction and the behaviour of delete and clear keys.

Monte Carlo methods use a random process to explore a state space. To analyse a user interface, the Monte Carlo process generates random key presses that control the user interface exactly as a user operating it would.

To use Monte Carlo for analysing numeric user interfaces, we choose a random number as the target *n* for the simulated operator to enter. A standard algorithm converts *n* to a sequence of keystrokes. This sequence of keystrokes is then modified by random processes to simulate well-known forms of human error, such as digit repetition. With a given probability, the simulated operator will notice such errors and correct them, e.g. by pressing the 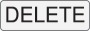 key. On completion of entering the modified sequence of keystrokes, the number actually entered is compared to the target value *n*.

Once a Monte Carlo experiment is set up, there is no overhead in performing experiments—an advantage over the costs of conventional user studies: recruiting participants, briefing them and collecting data. A typical Monte Carlo experiment can run continuously much faster than the fastest human can achieve in their best bursts of productivity. A Monte Carlo experiment is trivial to conduct, and designers can rapidly compare many designs. Finally, Monte Carlo experiments can be parametrized to study a range of behavioural patterns.

Ideally, delete keys should work adequately for repairing the majority of errors, and if the higher level cognitive process notices an error, pressing a clear key or following other strategies can be used to recover.

An operator can make a typing error by:

**repetition of a key**—repaired by pressing delete;
**omission of a key**—repaired by typing the missing key;
**transposition of two keys**—repaired by deleting two keys then retyping them in the correct order;
**substitution of one key for another**—repaired by pressing delete, then retyping the correct key; or by
**insertion of another key**—simply repaired by pressing delete.

These are *typing* errors and do not cover the possibility that the operator is mistakenly *intending* to type the wrong number, for instance following a *reading* error or *misunderstanding* how numbers work [2].

For the Monte Carlo model in this paper, we assume the errors occur independently of each other and with equal probability per keystroke, comparable to empirical results in [2].

We assume that once an error occurs and is noticed by the inner cognitive process that the operator continues as if the repair succeeds. It makes little difference whether the less than or equal to 4 or so repair keystrokes are themselves subject to error; modelling repair perfectly would require additional parameters (certainly, different repairs, being of different lengths, would have different overall error rates), and hence more ways of generating parameter-dependent results that might be misleading if they were estimated incorrectly. When assessing safety, the fewer assumptions and the fewer interactions between them, the better.

#### 3.2.1. Executable systems

A computer generates a Monte Carlo process and that controls the user interface. Hence to use a Monte Carlo method an executable system is required. If we were the developers of the systems we are analysing, this would be easy.

The approach is a *black box* approach, in that only a running (executable) version of the user interface is required, perhaps through only an API. The exact implementation (e.g. the program source code, which may contain intellectual property) is not needed, though source code would be convenient for using the technique to help improve the user interface.

In this paper, however, we carefully reverse engineer commercially available designs to obtain executable programs, one for each design we consider. Reverse engineering would not be necessary with collaboration from manufacturers or designers, but for number entry interfaces the task is not difficult.

We note that some number entry user interfaces are defective in complex and subtle ways, and for them reverse engineering serves to help expose their design problems [31].

#### 3.2.2. Excluded issues

The Monte Carlo implementation used here assumes that the operator can key an unlimited number of digits. Thus, in this paper we do not consider possible length or value restrictions on numbers, for example that (as happens on some real systems) no more than three digits are permitted or values no more than 999 are permitted.

Real designs typically do have limits, and the limits themselves may induce serious problems. Such limits will typically induce more error. One example of the significance is where a bank customer lost $100 000, reported in [32], and there are many other examples in common devices [18,33]. An example, specifically affecting decimal points, is the Baxter Colleague infusion pump: when the operator keys a number larger than 99, the Colleague ignores the decimal point key, hence 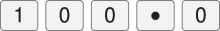 is treated as 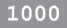, 10 times larger than the operator intended [4].

On all devices tested here, the delete key fails to work correctly when too many digits have been entered by the operator—and the user is not warned, so ironically correcting a known error (too many digits) creates another error (deleting other digits).

Many user interfaces that are used to enter short numbers scroll digits, so the number entered is made up of the most recent digits entered. This style of interface is often used for PIN passwords (e.g. for burglar alarms), typically of four or so digits—the approach allows the user to correct any error by simply re-entering the four digits of their PIN (strictly, an error in a four digit PIN can be corrected by at most four digits: if the user intends 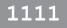 but enters 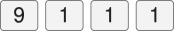, this error can be corrected by pressing 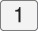 just once). This form of correction is not considered in this paper.

Many user interfaces have additional ways of correcting operator input. This paper only considers deletion and starting again (cancel). Alternatives include the use of arrow keys, insertion and overwrite modes, and more [34]. All of these features could be evaluated using the methodology introduced in this paper, but the number of design combinations grows exponentially and would unfortunately be unsuitable to present in a single paper. Note that as the number of error-correcting features increases, the number of strategies available to correct error also increases, and more empirical evidence is needed to inform how the operator selects between those strategies [31].

Many user interfaces have more keys than are necessary for entering numbers, as occurs with QWERTY keyboards. What should a user interface do when an operator presses a key that is not numeric? If the interface ignores the key, then what should the 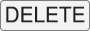 key do? If the number display is formatted to be more readable—e.g. following ISO standards, grouping digits in threes or following NHS guidelines (groups of 3 and 4, which is non-standard) thus apparently inserting spaces or commas—what should the user interface do when the user keys the separators? Under NHS guidance [35], it is mandatory to ignore the operator keying separators *and* mandatory to display spaces between groups of digits, as if the operator had entered them—which seems confusing, because if an operator keys a space it is ‘ignored’ yet one also appears in the display! The NHS standard fails to say what happens when an operator presses space in the middle of a group of digits: it is then unlikely to be wise to ignore it when it ought to trigger a warning. For the purposes of the present paper, all such design issues should be recognized as raising serious questions that need addressing empirically before designing dependable or safety critical systems. As such, evaluation of these choices is, in the first instance, beyond the scope of this paper.

Good practice is to provide key press feedback, such as a click. On devices where there is no feedback, the operator has no confirmation whether the key press was processed. On the Baxter Colleague, pressing keys rapidly will lose keystrokes, but there is no difference in key click feedback, because there is none before or after keystrokes are lost. Worse, when the infusion pump is not infusing, it beeps at intervals. If entering numbers in this mode, these beeps can coincide with a lost keystroke, thus misleadingly confirming the key was processed when in fact it was not. Our Monte Carlo models do not consider keystroke feedback.

This paper has only space to evaluate a few common designs; there are many ways to implement number entry features idiosyncratically, and it is impossible to compare all of them in this paper. One example will be sufficient to illustrate some of the types of issue that may be encountered. On the Samsung Android (v. 2.3.3, 2014), pressing 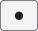 gets displayed as 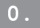, that is, the Samsung inserts a leading zero the operator did not key. Hence (though Samsung could have designed it differently) pressing 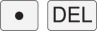 does not result in nothing, but in the digit zero. The difference between these results can be exposed by the operator continuing after the correction: 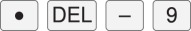 becomes 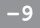, but 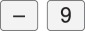 becomes 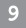, even though the operator might consider the two key sequences to be exactly equivalent.

There are no problems, in principle, in using the Monte Carlo method to evaluate such designs, it is just impractical to cover so many design variants in a single paper.

Finally, number entry is usually part of a larger task, such as entering figures into a spreadsheet, in turn itself part of a larger task such as performing statistical analysis of an experiment, or calculating radiation therapy doses, or completing financial returns for taxation. For all such tasks, there are generally additional methods (beyond the scope of the present paper) for checking and correcting data, for instance by using double entry, plotting graphs to identify outliers or using numbers with special properties, such as check-digits. How the operator validates data can have a huge impact on the quality of results; for example, in data entry experiments [36], visual checking resulted in 30 times more errors than double entry.

#### 3.2.3 Experiments comparing eight designs

We compare four common commercial designs (we abbreviate with the letters ABCN) with four new designs (DEFG). It is important to emphasize that the functionalities of these designs are equivalent—on all designs, users can enter and correct numbers, and apart from infrequent cases (e.g. deleting decimal points) the designs are indistinguishable. Few operators would be able to tell the designs apart yet, as we shall show, their induced error rates are different.

The designs explore various features, as below. See table 3 for a concise summary of the designs, and appendix C for a formal description of the designs. (Short names are used in figures and tables to save space.)

**Table 3.** Summary of designs. ABCN are common, commercial designs; DEFG are proposals. Some unusual defective designs [17] are not considered here. Table 4 illustrates the designs on example keystroke errors and recoveries. Appendix C provides specifications of the designs, sufficient for them to be implemented.

design	brief description
A	delete key ignores decimal points, and the design ignores multiple decimal points. Thus 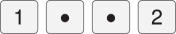 and 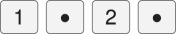 are both equivalent to 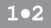 ; 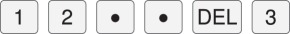 is equivalent to 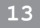 ; and 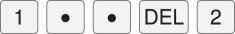 is equivalent to 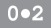 . Design A occurs in many systems and devices such as the Casio HR-150TEC, Hewlett Packard EasyCalc 100, etc.
B	delete key works correctly, but the design ignores multiple decimal points. Thus 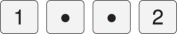 is equivalent to 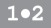 (as in design A), but 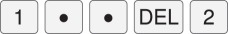 is equivalent to 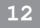 (60 times higher than in design A). Design B occurs in many devices, such as the Samsung Android, Apple iPhone, etc.
C	correct design, exemplified by the Casio fx-85GT and many familiar keyboard-based applications on PCs, such as Microsoft Word
D	correct design, which also intercepts key bounce. A number entered with a repetition is blocked, and the operator has to re-enter it
E	correct design, which also checks ISMP recommendations. Invalid numbers are intercepted and the operator retypes them, possibly making further errors
F	correct design, which also enforces ISMP recommendations and ensures the number is correctly entered
G	correct design, which also enforces value to be within a factor of 5 of the intended number
N	no delete key. Noticed errors corrected by clearing and starting over
—	we know of no design that implements delete incorrectly but which implements decimal point correctly

**Design A** Many designs always display exactly one decimal point, even if the operator has typed none or several. On such designs, the 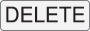 key only deletes digits, probably because deleting decimals is problematic.

Design A short name: **Broken delete & decimals**.

**Design B** More sophisticated designs show a decimal point only if the operator has in fact entered one, but they will still only show at most one decimal point. The 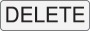 key deletes digits and the decimal point, but obviously keying 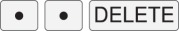 will not have the desired effect as the second decimal point was never displayed.

Design B systems ignore a second or subsequent decimal point, although it would also be possible to move the decimal point to the far right of the number displayed. We do not consider this design variation in this paper.

Design B short name: **Fixed delete only**.

**Design C** Correcting the design defects in designs A and B but with no other features produces design C. Digits and decimal points are treated equally, and the 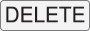 key deletes them both. Multiple decimal points can be keyed, which implies an operator's input may be invalid and rejected by the design, thus forcing the operator to correct it.

Design C short name: **Fixed delete & decimals**.

**Design D** We know that key bounce is a serious design problem [37]. Design D forces all repetitions, even in intended numbers like 100, to be entered twice. Design D may cause occasional extra work for users, but it effectively blocks errors from key bounce.

Design D short name: **Debounced**.

**Design E** Designs E and F enforce Institute of Safe Medication Practices (ISMP) recommendations [38].^2^ In both designs E and F, when a number fails the ISMP test, the user must start again. Note that (in contrast to design D that rejects repeated keys) *all* numeric values can be expressed as valid ISMP numbers.

In design E, when a number fails the ISMP test, the operator must re-enter it, possibly making further errors.

Design E short name: **ISMP**.

**Design F** Design F simulates optimal performance for design E. In effect, after detecting a non-ISMP number, design F cues the operator to employ higher level processes to re-enter the number more carefully and hence correctly: e.g. interrupting lower level cognitive processes so higher level processes take thoughtful action [33].

Note how the Monte Carlo experiments need not explore *how* a human operator would really interact: examples like design F show that hypothetical user interaction can also be evaluated. Put another way, design E is a real user interface design, and design F provides the most optimistic behaviour for that design for evaluation purposes.

Design F short name: **Low bound ISMP**.

**Design G** Design G enforces range checking, like a hard limit on a dose error reduction system [39], requiring entered numbers to be within an illustrative factor of 5 of intended numbers.

Design G short name: **Range check**.

**Design N** Finally, it is interesting how well a design with *no* delete key might perform. Hence, we consider design N, which has a 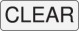 key but no 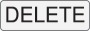 key (or the operator is trained not to use any delete key). We know of no design that implements 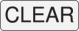 defectively.

Design N short name: **No delete (clear only).**

We could of course continue generating design combinations indefinitely, for instance combining design N with ISMP checking. In our previous paper [5], we only evaluated designs with neither delete nor clear. Once a Monte Carlo test bed is set up, performing such experiments and comparing design variations is easier than describing them.

#### 3.2.4. Number of Monte Carlo tests

We performed 10^8^ Monte Carlo experiments per design (i.e. simulating keying in 10^8^ numbers on each of the 8 user interface designs) measuring risk with vulnerability set at 100 different values, 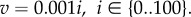


## 4. Results and discussion

### 4.1. Results

As expected, our experiments show risk increases with increasing vulnerability. The relation for all designs is linear, though the intercepts for designs AB have non-zero risk for zero vulnerability; this is strong evidence that these common designs are defective.

All designs have linear regression coefficient of determination (correlations) *R*^2^ ≥ 0.9906.

**Figure 1.**
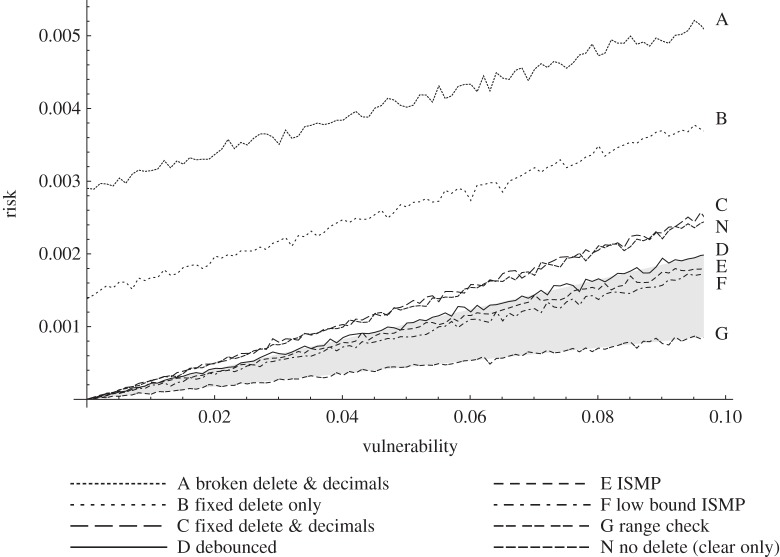
Risk against vulnerability for different designs (figure 2). Risk increasing with vulnerability is expected (lower lines/gradients are safer), but different designs perform differently. Defective designs AB have potentially unacceptable risk even for ‘perfect’ (*v* = 0) operation; the alternative designs prove such risk is avoidable. The grey region covers designs that additionally cue the operator to manage error; all are safer than conventional designs.

Figure 1 exhibits results graphically. Designs A and B are worse, and have non-zero risk at zero vulnerability. Design N, with no delete, performs better than devices with a defective delete; it performs marginally better than the correct design C because at most one noticed error can occur per number. When designs aid the operator detecting error (DEFG) risk is further reduced.

**Figure 2.**
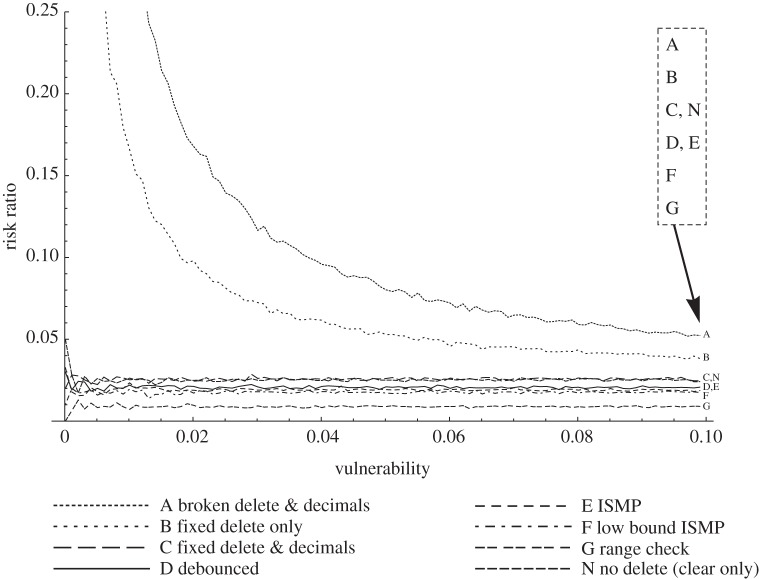
Risk ratio, the ratio of risk divided by vulnerability; compare visualization with figure 1, which is the same data. The distinctively defective designs A and B stand out. They counter-productively make risk ratio increasingly worse as the operator tries to reduce vulnerability: that is, however vigilant the operator (reducing *their* vulnerability, even to zero) the design defects ensure there is still residual risk (so the risk ratio goes to infinity). Put another way, even a perfect operator might be blamed for the problems these poor designs themselves are creating.

See figure 2 caption for a discussion of risk ratio results for the designs considered.

### 4.2. Discussion

The analysis showed that two designs, A and B, are clearly not suitable for safety critical contexts. The analysis also shows that improvements can be achieved by addressing the faults A and B illustrate with the refinements of the other designs.

In all cases, simple tests could be readily employed on seeing a system that would provide a diagnostic test of which design the device was. In particular, anyone procuring interactive systems or devices could determine an A or B design within seconds of the device being switched on and tested. Hopefully, they would reject such designs equally quickly. (Tables 3 and 4 give concrete examples, and appendix C gives design rules that will help distinguish one design from another.)

**Table 4.** Delete key behaviour. Astonishingly, many numerical user interfaces always show a decimal point even if one has not been keyed (regardless of the delete key). For clarity, the right-hand column only shows a decimal point if it has been keyed and not deleted. It matters: if the display always shows a decimal point, if the next keystroke is a 0, it *unpredictably* leaves the number unchanged or multiplies it by 10. (This table was generated automatically by the Monte Carlo simulation program: hence what it describes is what was evaluated.)

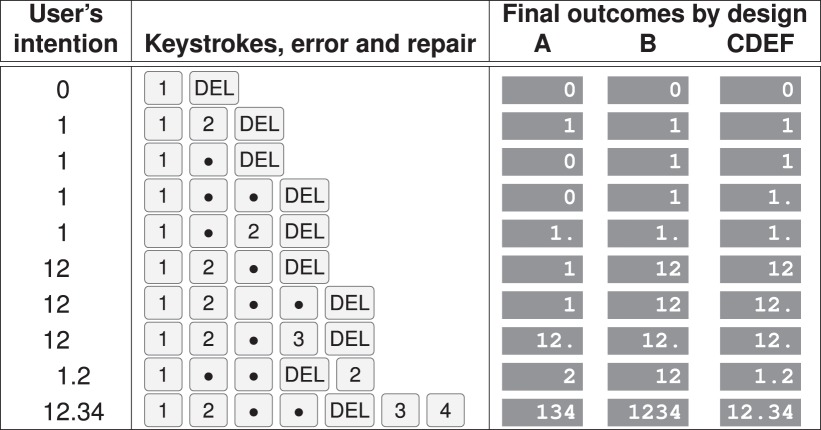

The best performing Monte Carlo models assume the design provides feedback to the operator to influence their behaviour to manage errors. This was an assumption behind designs F and G. Error warning messages are often transient in nature and can easily be missed by operators. Our eye tracking experiments [2] show operators devote more and longer eye fixations on the keyboard than on the display, so therefore warnings in the display, particularly transient information, are likely to be missed.

The Monte Carlo experiments show that error should be detected, and if it is, further risk can be reduced. However, the system detecting error and the operator realizing error has been detected and taking action are different things. Errors have to be clearly announced to the operator, and this typically means latching the warning messages so that they are still visible when the operator looks for a result following their actions. On a calculator, the natural place is the number display, where calculators conventionally report answers. On other devices, other locations (or sounds or physical feedback, like vibration) may be used. But if the operator does not know an uncorrected error has occurred, they are induced to continue and the consequences of the error will escalate rather than be mitigated.

#### 4.2.1. Recommendations

Our results show that poor user interface designs perform much worse for number entry than better-designed user interfaces. Unfortunately, until performance figures are published, it is very hard to know what is preferable when choosing between manufacturers' products.

(1) Monte Carlo methods are easy to use and reveal design flaws in user interfaces very effectively. In number entry user interfaces, evaluation can be easily quantified. Monte Carlo methods can be used to rank user interface designs for safety.
(2) The safest general-purpose number entry system is design E, and other designs show that more context (e.g. design G) can further increase safety. If the ISMP number syntax is felt to be intrusive for the application (although it imposes no numeric limitations) then design C may be preferred.
(3) In the absence of evidence of correct design and implementation, prefer systems (like design N) with no delete key.
(4) Train operators to use 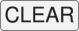 (or equivalent) instead of 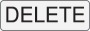. Human factors specialists may be used to help seek ways to help teams be more resilient when using defective systems that are already in use.
(5) Use our specifications of the various designs (appendix C and the examples in tables 3 and 4) to try to establish which design is being considered. Our results may then give an estimate of the relative performance of the designs being considered. Our analysis and results suggest that designs A and B are misleading and unsafe.
(6) The question may arise, ‘The new designs are better, but are they better enough?’ An investment in evaluation at the design stage, as suggested in this paper, can provide improvements to user interfaces, which however small, will benefit users indefinitely into the future. Some of those benefits may include avoiding catastrophes, which will amply repay the marginally increased effort for the designers. See the note on technical debt in appendix A.

#### 4.2.2. Little need to measure vulnerability empirically

Since none of the best fit lines intersect, the best designs are best *regardless* of vulnerability. For practical purposes, the ranking of design quality is independent of vulnerability.

This result is important because the empirical evaluation of user interfaces is very time-consuming, can only be performed after a design has been created, and is very difficult to design to cover enough errors to be statistically significant (operator error rates are typically very low). Moreover, it is unreliable to generalize laboratory experiments to provide estimates for the real-world situations where the systems will be used.

One might wish to estimate vulnerability to estimate the improvement that can be achieved by replacing one design by another. However, using Monte Carlo methods to develop and evaluate design variations can help inform A/B tests, which will be more reliable to perform than experiments to measure vulnerability directly.

### 4.3. Conclusions

We have shown that number entry systems, and hence user interfaces more generally, are a rich source of scientific investigation—we would argue comparable to biological species or archæological artefacts, say. Unlike conventional objects of science, however, number entry systems do not stand apart from the observer, and indeed the nature of human error makes studying number entry both problematic and fascinating, since it occurs in design, in use and in observation. While the development of number notations has been refined over centuries [40,41], the new field of ‘interactive numbers’ has yet to be developed [42].

Errors cannot be avoided; to err is human. However, many design errors can be eliminated, and operators should always be warned (or pre-warned) appropriately if the nature of the error cannot be correctly handled and repaired, for example, if there is a limit (such as the maximum number of keystrokes) the operator has exceeded.

It was an insight in the 1940s to argue that focusing on operator error was inadequate [14]. The *whole* system fails to appropriately manage errors: the operator is no more the cause of any error than the design. Indeed, design error is ubiquitous—it is astonishing that designs with non-zero risk for zero vulnerability persist in the market. This paper will help designers, system purchasers (whether procurers or consumers) and users be more critical, particularly about number entry tasks.

Design error is hard to notice because designers lose situational awareness and because operators take designs for granted, assuming technology is good and newer technology is better. In fact, there is considerable variation in design quality, even for equally new designs. This paper showed that identifying and fixing design error can have a more strategic impact than training operators to be more vigilant, whether in standard operating procedures or human factors more generally. Given that normal error-free operator behaviour cannot distinguish between the designs, little training if any is required to take advantage of the possible improvements.

## Supplementary Material

Please see http://www.harold.thimbleby.net/montecarlo

## Endnotes

1 If there is no  key shown on the keypad, pressing  will change it to be displayed as . There is no  key as such, but deleting on the iPhone is achieved by swiping a finger left or right across the number display (if it was a number the user entered, rather than the result of a calculation).

2 An ISMP number forbids ‘naked decimal points’ such as .1 (potentially misread as 1) and does not allow trailing zeros after a decimal point, as in 1.0 (potentially misread as 10). Additional criteria used here that ISMP fail to state: the number must have at least one digit, at most one decimal point, and must not start with 0 unless the next character is a decimal point.

## Data accessibility

See the online version of this article (http://dx.doi.org/10.1098/rsif.2015.0685) for the electronic supplementary material, including a Mathematica 10 notebook (also available in PDF) explaining and generating all models used in the paper. All data are available, in CSV (simple text files of comma separated values), Microsoft Excel and Mathematica formats.

## Appendix A. Why do design problems persist?

Section 2.4 refers to this appendix.

(1) What this paper calls *defects* may be dimissed as trivial. The word ‘trivial’ is equivocal (trivial = easy to ignore; trivial = easy to fix).
(2) Users can be blamed—and blame themselves—for error. Error-inducing design can create additional income. Some common ATMs (cash machines) display  and as digits are keyed, the number scrolls in from the right—so the first two digits, say , appear as a fraction (in this case, ) and to get an amount the ATM can dispense, the operator must finish with two consecutive zeros. This unnecessary design complexity is ‘fail safe’ in that an ATM cannot dispense coins, but if the user wanted $500 they might only get $5, and perhaps pay a fee to get it, and another fee to get the $495!
(3) Technical debt [43] describes the savings made by developers which users pay off in the future. The cost savings at development time mean developers often discount user interface design issues, especially ones thought ‘trivial’ and which have little impact on short-term business; the ATM example, above, is a concrete case of actual debt affecting users effectively paying off the consequences of saved effort during development.
(4) Many think ‘reading a number is program code anyone can write; it is a few lines of code and will obviously work’. Neither rigorous testing nor formal development seems necessary for such a seemingly simple problem.
(5) Uncorrected errors in user interfaces occur because we do not notice them. If we do not notice them, then it is likely that related bugs in user interfaces are not noticed either. This is a vicious circle: error handling in user interfaces is very poor.
(6) Confirmation bias is the tendency of people to confirm their beliefs, to prefer to check things they think are right. We rarely notice our errors (if we noticed them, we would not make errors), so we tend to notice our successes and ignore our errors and the design errors that create them.
(7) Error is very hard to research^3^ and has little presence in the user interface design literature. For example, the classic book on the science is Card, Moran and Newell [44] which specifically excludes human error; it is concerned only with skilled, error-free human performance. Norman [45] is one of the very few papers mapping the psychology of error into practical design advice.
(8) Most of the user interface design literature ignores the programmer, and thus programmers build user interfaces but have negligible awareness of human factors. Landauer [46] is a classic book promoting user-centred design, yet its model of development is user-centred design then ‘just’ tell the programmer what to do. A notable exception to the trend is Thimbleby [7].
(9) Rigorously developed systems must be traceable back to clear requirements. Number entry is typically a requirement in itself that is not decomposed into further requirements; the details of individual key presses are considered trivial and not formalized. A case in point, the ISMP requirements for safe number formats has critical oversights this paper identifies (see endnote 2).
(10) While there are many programmers, only a very low proportion can program well. Appendix B exhibits a publicly available proposed worldwide Web standard for parsing numbers, and as the appendix points out, it accepts (without reporting errors) invalid numbers like 1.2.3 and 2E3.2 (presumably 2 × 10^3.2^ but actually parsed as 2 × 10^3^, since the parser terminates prematurely at the unexpected decimal point).
  It is invidious to select examples, but we chose the example exhibited in appendix B because the worldwide Web has one of the largest user bases of any system, and therefore the advantages of good requirements and specification are obvious (the number specification was also made public, which was a necessary criterion for review in this paper).
(11) Serious, high-profile problems, like the 22-year-old ShellShock bug in bash (disclosed in 2014) share similar problems: ShellShock exploits bash's incorrect parsing of trailing strings, a problem identical to one of the number parser problems exhibited in appendix B. In other words, bad programming is common; the design defects reported in this paper share themes with other widespread bugs.
(12) While poor security practices are taken seriously, poor-quality user interfaces are dismissed. Thus, Fu [47] reports on a security weakness caused by a buffer overflow problem—bad hackers may exploit this weakness, so it needs fixing; yet the same buffer overflow problem in a user interface [32] is ignored—why would good operators want to exploit bugs [48]?!
(13) There is effectively no professional regulation controlling practice in the software industry. Anybody can program anything.
(14) Nobody provides assuredly better systems. The state of the art in computing (particularly consumer devices) is driven by excitement, not by dependability.
(15) When errors do occur that cause harm, often the operator is blamed. Indeed, when devices have regulatory approval, it is almost inevitable that operators are blamed because (in some jurisdictions) regulatory approval implies the design is fit for purpose, and therefore any faults in use must be due to the operator.
(16) Software warranties typically argue that the developers are not responsible for any problems experienced in the use of the system [49]. If nobody takes responsibility for software quality and denies liability for defects, why would manufacturers invest in unnecessary quality that does not improve sales? Some warranties argue ‘by using this software the operator agrees…’ and may also include caveats such as ‘the operator must exercise their own judgement to interpret results’—which begs the question why anybody would want to use critical systems that cannot be relied upon!
(17) Procurement is generally driven by cost not safety, and in any case, safety for many systems is not quantifiable.
(18) Because software quality is poor and it is not easy to measure quality, regulators are in the impossible bind that, on the one hand, the market does not demand higher quality, and on the other that if higher quality was a regulatory requirement many products the market finds valuable would have to be phased out. An overwhelming ‘regulatory burden’ that *appears* to offer negligible benefit to manufacturers is not going to be pursued.
(19) Until the present paper, there are no effective tools or processes for finding and quantifying user interface design problems, particularly problems that have been overlooked in requirements.

## Appendix B. W3C floating-point numbers

Appendices A and C refer to this appendix.

The following code was copied from the WorldWide Web Consortium's *A vocabulary and associated APIs for HTML and XHTML W3C Working Draft* [50]. This code is notable because it is presented by a leading organization with a worldwide impact, but what is presented as a computer program is—we argue—in fact a list of vague English instructions, with misleading sophistication and pedantry.

It is hard to read and hard to reason about. It is notable for not using assertions or other standard features for helping assure quality, let alone giving the requirements it should implement. It is not presented with unit tests. Clearer approaches have been suggested elsewhere [51].

This W3C specification fails completely to define *how* an operator interacts with numbers—and thus raises many design issues it fails to discuss, such as what happens when an operator keys a number that is ‘too long’ and perhaps is truncated so displaying a misleading number. Appendix C (based on the notation developed in [3]), which defines the designs tested in this paper, illustrates how simply interaction can be specified.

The original code is presented, followed by a non-exhaustive but representative list of more specific criticisms relevant to the concerns of this paper.

(1) Let input be the string being parsed.
(2) Let position be a pointer into input, initially pointing at the start of the string.
(3) Let value have the value 1.
(4) Let divisor have the value 1.
(5) Let exponent have the value 1.
(6) Skip whitespace.
(7) If position is past the end of input, return an error.
(8) If the character indicated by position is a U+002D HYPHEN-MINUS character (–):
  (a) Change value and divisor to –1.
  (b) Advance position to the next character.
  (c) If position is past the end of input, return an error.
(9) If the character indicated by position is not one of U+0030 DIGIT ZERO (0) to U+0039 DIGIT NINE (9), then return an error.
(10) Collect a sequence of characters in the range U+0030 DIGIT ZERO (0) to U+0039 DIGIT NINE (9), and interpret the resulting sequence as a base-10 integer. Multiply value by that integer.
(11) If position is past the end of input, jump to the step labelled conversion.
(12) If the character indicated by position is a U+002E FULL STOP (.), run these substeps:
  (a) Advance position to the next character.
  (b) If position is past the end of input, or if the character indicated by position is not one of U+0030 DIGIT ZERO (0) to U+0039 DIGIT NINE (9), then jump to the step labelled conversion.
  (c) Fraction loop: multiply divisor by 10.
  (d) Add the value of the character indicated by position, interpreted as a base-10 digit (0.9) and divided by divisor, to value.
  (e) Advance position to the next character.
  (f) If position is past the end of input, then jump to the step labelled conversion.
  (g) If the character indicated by position is one of U+0030 DIGIT ZERO (0) to U+0039 DIGIT NINE (9), jump back to the step labelled fraction loop in these substeps.
(13) If the character indicated by position is a U+0065 LATIN SMALL LETTER E character (e) or a U+0045 LATIN CAPITAL LETTER E character (E), run these substeps:
  (a) Advance position to the next character.
  (b) If position is past the end of input, then jump to the step labelled conversion.
  (c) If the character indicated by position is a U+002D HYPHEN-MINUS character (–):
    (i) Change exponent to –1.
    (ii) Advance position to the next character.
    (iii) If position is past the end of input, then jump to the step labelled conversion.
      Otherwise, if the character indicated by position is a U+002B PLUS SIGN character (+):
  (a) Advance position to the next character.
  (b) If position is past the end of input, then jump to the step labelled conversion.
  (c) If the character indicated by position is not one of U+0030 DIGIT ZERO (0) to U+0039 DIGIT NINE (9), then jump to the step labelled conversion.
  (d) Collect a sequence of characters in the range U+0030 DIGIT ZERO (0) to U+0039 DIGIT NINE (9), and interpret the resulting sequence as a base-10 integer. Multiply exponent by that integer.
  (e) Multiply value by 10 raised to the exponentth [sic] power.
(14) Conversion: Let *S* be the set of finite IEEE 754 single-precision floating-point values except −0, but with two special values added: 2^128^ and −2^128^.
(15) Let rounded-value be the number in *S* that is closest to value, selecting the number with an even significand if there are two equally close values. (The two special values 2^128^ and −2^128^ are considered to have even significands for this purpose.)
(16) If rounded-value is 2^128^ or −2^128^, return an error.
(17) Return rounded-value.

Comments on the W3C algorithm:

(1) The W3C algorithm permits number entries such as 2E3.2, 1.2.3 and so forth, without reporting an error. A key problem is that the step labelled ‘conversion’ does not check that string parsing has been completed, and therefore unexpected characters beyond the ‘end’ of the number are ignored. Numerous misleading examples can be imagined, such as 1+1, 2E–4 and so on, as well as culturally plausible errors such as 1,2 (intending 1.2) which is read as 1.
(2) The conversion assumes IEEE 754 single-precision floating-point *binary* format (binary32), which covers 2^−126^ to 2^127^, and supposes that the arithmetic conversion is exact, then rounds to the set *S* or the overflow values. It is unfortunate that the ‘return an error’ does not distinguish between a syntax error and a well-formed number that happens to have numeric overflow. Since the binary format allows 7.22 decimal digits with an exponent at most 38.23, reading a decimal number (as here) would be better handled using the IEEE 754 decimal floating-point standard (decimal32).
(3) The IEEE standard caters for returning NaN [26] as well as ±∞, which may offer better ways of handling overflow than by the W3C indiscriminate ‘error’. However, a common problem in programming is detecting an error in the ‘wrong’ place, and merely ignoring the error elsewhere; ideally, the W3C standard should discuss error handling, and the parsing of numbers should support or be consistent with that approach.
(4) The W3C algorithm attempts to detect overflow, in the sense of parsing a number outside of the IEEE single-precision range, but the approach taken is flawed, as it assumes the calculation itself does not overflow. The algorithm permits any integer exponent without detecting overflow; parsing the likes of 1E1000000 … can overflow many implementations.
(5) The bound checking uses –2^128^ < *n* < 2^128^; yet these bounds have little significance to users—the algorithm is in base 10 not base 2! Had the valid range been ±10^38^ (10^38^ is the largest power of 10 no more than 2^128^) the code would have been much easier to implement correctly, since the bound can be checked by simply counting (decimal) digits.
(6) While the algorithm discusses overflow, it fails to detect or manage display overflow—for example, if a user keys more digits than fit in a display box, the result is a misleading overflow, but is not detected by this code.
(7) In the context of this paper, it is interesting that this proposed code does not specify any error correction (what would a  or  key do?) as this is left entirely to the browser (or the user's operating system), for which there are no standards.

## Appendix C. Design specifications

Section 3.2.3 and appendix B refer to this appendix.

Numeric user interfaces can be considered implemented with a string buffer, to which the user's keystrokes are normally appended. Hence the ‘last’ digit or character in the buffer is the rightmost character.

The delete key normally deletes the last key in the buffer. We can specify the behaviour of the buffer by a precondition, and a postcondition that applies if the precondition was true. If no precondition is true, nothing happens. Note that some actions (e.g. press delete) may have several rules, depending on the contents of the buffer, and (as design D shows when pressing a digit) multiple rules may all apply in a single case. We use the declarative notation from [3] but we use English to describe the conditions intuitively without introducing further formalism.

Rules are written in the following form, numbered for convenient reference:

When an action occurs and the precondition is fulfilled, the postcondition is achieved (in some way by software that we do not need to discuss here). The same action may need several rules, so preconditions cover different eventualities. For example,

…is a pair of rules specifying that the delete key deletes the last digit, but because the buffer (for this design) is not allowed to have no digits, when the buffer only contains one digit, it is not ‘deleted’ but made to be zero. As a special case, if the buffer was zero, then it will still be zero after pressing delete. (These simple illustrative rules say nothing about behaviour with decimals.)

Conditions may refer to ‘full buffer’, which means the number of digits in the buffer is the maximum permitted by the device, perhaps eight characters. In many designs showing a decimal point does not affect the buffer limit, since each character in the buffer has an optional decimal point—which is a design decision that of course makes it impractical to display more than one adjacent decimal point. Our definitions below ignore the user keying additional digits when the buffer is full; arguably better designs would alert the user and ‘lock up’ until  is pressed to clear the display (this paper did not evaluate the effect of buffer overflow).

We note that the two defective designs (A and B) have longer descriptions than the other designs. This suggests that designs A and B were not specified declaratively but, for instance, as side-effects of running an imperative program, so the special cases our notation makes explicit were probably never considered by programmers. It is also noteworthy that the specification of W3C number input in appendix B is imperative in exactly this way—it is very hard to infer the rules the program implements just by reading the program, even when helped by the comments.

**C.1. Design A: broken delete & decimals**

Design A occurs in many systems and devices such as the Casio HR-150TEC, Hewlett Packard EasyCalc 100, etc. The display always shows exactly one decimal point.

Examples: delete key ignores decimal points, and the design ignores multiple decimal points. Thus pressing  and  are both equivalent to  is equivalent to ; and pressing  is equivalent to .

**C.2. Design B: fixed delete only**

Design B occurs in many devices, such as the Samsung Android, Apple iPhone, etc. Delete key works correctly, but the design ignores multiple decimal points. The display can show zero or one decimal points.

Examples: pressing  is equivalent to  (as in design A), but pressing  is equivalent to  (60 times higher than in design A).

**C.3. Design C: fixed delete & decimals**

Nominally correct design, exemplified by the Casio fx-85GT and many familiar keyboard-based applications on PCs, such as Microsoft Word. The display can show zero, one or multiple decimal points.

**C.4. Design D: debounced**

Correct design, which also intercepts key bounce. A number entered with a repetition is blocked, and the operator has to re-enter it.

Design D is design C, but with this rule added:

and this rule replacing C.2:

Since pressing enough  keys is equivalent to pressing , then ‘since last cleared display’ in the rules above more precisely means ‘since the display was last ’.

**C.5. Design E: ISMP**

Correct design, which also checks ISMP recommendations. Invalid numbers are intercepted and the operator retypes them, possibly making further errors.

Similar to design D, except the added rules are from ISMP. Rather than rejecting repetition, ISMP rejects numbers with a leading zero if the number greater than 1; no leading zero if number less than 1; trailing zeros after a decimal; decimal if no digits after it; a trailing zero after a decimal; more than one decimal.

Since entering a valid number like 1.05 would briefly break the ISMP rule (when keying the 0, which appears to be a trailing zero), the ISMP rule is checked only when entering the number is completed.

**C.6. Design F: low bound ISMP**

Correct design, which, like design E, enforces ISMP recommendations but ensures the number after an operator error is always correctly entered. Design F therefore gives a lower bound on the effectiveness of the ISMP intervention—it behaves as if number entry is perfect (after detecting an operator error).

**C.7. Design G: range check**

Like design C, except that the condition is that a number entered more than 5 × *n* or less that *n*/5 is barred the first time it occurs.

Design G is a nominally correct design, which also enforces value to be within a factor of 5 of the intended number. Although 5 is an arbitrary choice, chosen for this paper, in a typical dose error reduction system, a fixed range is set depending on the intended therapy—effectively, selecting the drug sets the range, whereas in this paper the range is set as a proportion of the intended number.

A dose error reduction system will also have ‘soft’ and ‘hard’ limits. Design G has ‘soft’ limits—a warning occurs, and the user can then re-enter the number. A hard limit, in contrast, cannot be over-ridden.

**C.8. Design N: no delete (clear only)**

Like design C, but without any delete key. When the operator notices errors they must be corrected by clearing and starting over. Like design C, and unlike designs DEFG, N does not detect any errors.
