# Supplementary material for: Unreliable numbers: error and harm induced by bad design can be reduced by better design
Source: J R Soc Interface. 2015 Sep 6;12(110):20150685. doi: 10.1098/rsif.2015.0685 (PMC4614478; doi:10.1098/rsif.2015.0685)
Supplement: Please see http://www.harold.thimbleby.net/montecarlo [file rsif20150685supp1.doc]

**Supporting online material**

See <http://www.harold.thimbleby.net/montecarlo> for supporting online material [for review and/or publication purposes], including a Mathematica 10 notebook (also available in PDF) explaining and generating all models used in the paper. All data is available, in CSV (comma separated values), Microsoft Excel, and Mathematica formats.
